# Towards understanding the impact of mycorrhizal fungal environments on the functioning of terrestrial ecosystems

**DOI:** 10.1093/femsec/fiaf062

**Published:** 2025-06-13

**Authors:** Olivier Nouwen, Francois Rineau, Petr Kohout, Petr Baldrian, Nico Eisenhauer, Natalie Beenaerts, Sofie Thijs, Jaco Vangronsveld, Nadejda A Soudzilovskaia

**Affiliations:** Centre for Environmental Sciences, Environmental Biology, Hasselt University, Campus Diepenbeek, Agoralaan, Gebouw D, 3590 Diepenbeek, Belgium; Institute of Biology, Leipzig University, Puschstr. 4, 04103 Leipzig, Germany; German Centre for Integrative Biodiversity Research (iDiv) Halle-Jena-Leipzig, Puschstr. 4, 04103 Leipzig, Germany; Centre for Environmental Sciences, Environmental Biology, Hasselt University, Campus Diepenbeek, Agoralaan, Gebouw D, 3590 Diepenbeek, Belgium; Institute of Microbiology of the Czech Academy of Sciences, Videnska 1083, 14220 Prague, Czechia; Faculty of Science, Charles University, Albertov 6, 128 43 Prague, Czechia; Institute of Microbiology of the Czech Academy of Sciences, Videnska 1083, 14220 Prague, Czechia; Institute of Biology, Leipzig University, Puschstr. 4, 04103 Leipzig, Germany; German Centre for Integrative Biodiversity Research (iDiv) Halle-Jena-Leipzig, Puschstr. 4, 04103 Leipzig, Germany; Centre for Environmental Sciences, Environmental Biology, Hasselt University, Campus Diepenbeek, Agoralaan, Gebouw D, 3590 Diepenbeek, Belgium; Centre for Environmental Sciences, Environmental Biology, Hasselt University, Campus Diepenbeek, Agoralaan, Gebouw D, 3590 Diepenbeek, Belgium; Centre for Environmental Sciences, Environmental Biology, Hasselt University, Campus Diepenbeek, Agoralaan, Gebouw D, 3590 Diepenbeek, Belgium; Department of Plant Physiology and Biophysics, Institute of Biological Sciences, Maria Curie-Skłodowska University, 19 Akademicka Str., 20-033, Lublin, Poland; Centre for Environmental Sciences, Environmental Biology, Hasselt University, Campus Diepenbeek, Agoralaan, Gebouw D, 3590 Diepenbeek, Belgium

**Keywords:** arbuscular mycorrhiza, ectomycorrhiza, ericoid mycorrhiza, Mycotron experiment, soil biochemical cycles, soil properties

## Abstract

Mutualistic interactions between plants and soil fungi, mycorrhizas, control carbon and nutrient fluxes in terrestrial ecosystems. Soil of ecosystems featuring a particular type of mycorrhiza exhibit specific properties across multiple dimensions of soil functioning. The knowledge about the impacts of mycorrhizal fungi on soil functioning accumulated so far, indicates that these impacts are of major importance, yet poorly conceptualized. We propose a concept of mycorrhizal fungal environments in soil. Within this concept, we discuss knowledge gaps related to the understanding and quantification of mycorrhizal fungal impacts. We introduce an experimental framework to address these gaps in a quantitative manner, and present the field experiment ‘Mycotron’, where we established vegetation series featuring three mycorrhizal types; ericoid (ERM), ecto- (ECM), and arbuscular mycorrhiza (AM), to quantitatively assess mycorrhizal fungal impacts on soil functioning. The experimental treatments entail manipulations in dominance levels of vegetation of three mycorrhizal types (AM, ECM, and ERM) in standardized soil conditions. This experiment constitutes a unique testbed to quantitatively evaluate the impacts of distinct mycorrhizal fungal environments on a large variety of ecosystem functions. Our approach aids the quantification of microbiota and plant–microbial interaction impacts on soil biochemical cycles.

## Teaser

We propose a mycorrhizal fungal environment concept to describe and quantify the impacts of mycorrhizal fungi on soil processes and introduce the new Mycotron common garden experiment to implement and test this concept.

## Introduction

### Mycorrhizas, their interactions, functioning, and diversity

Mycorrhizas are mutualistic relationships between plants and soil fungi featured by almost all terrestrial plant species (Brundrett 1991, Smith and Read 2008). This relationship enables plants to increase uptake of water (Ruth et al. 2011) and nutrients, such as phosphorus, nitrogen, and micronutrients (Smith and Read 2008). In exchange, plants supply fungi with photosynthates. This mutualistic relationship does not only affect the nutrition of the plants and fungi, but also governs many important soil functions (Tedersoo and Bahram 2019), as mycorrhizal fungi influence soil carbon sequestration, contribute to weathering of minerals (Finlay et al. 2009), protect the plant from biotic and abiotic stressors, decrease soil erosion, and promote soil aggregation (Genre et al. 2020).

Depending on the fungal and plant partner species involved, mycorrhizal symbioses are categorized into four mycorrhizal types, three of which are geographically most widespread (Cairney 2000). Arbuscular mycorrhizas (AM) are most abundant, found in 72% of vascular plants (Brundrett 2009, Brundrett and Tedersoo 2018, Soudzilovskaia et al. 2020), and geographically most common (Soudzilovskaia et al. 2019). Arbuscular mycorrhiza fungi (AMF) are also taxonomically monophyletic (Brundrett and Tedersoo 2018). In contrast, ectomycorrhizal fungi (ECMF) and ericoid mycorrhizal fungi (ERMF) are polyphyletic and form symbiosis with ~2% and 1.5% of plant species, respectively (Brundrett 2009, Wang et al. 2010, Field et al. 2015), Brundrett and Tedersoo 2018, Soudzilovskaia et al. 2020). Worldwide AM plants store 240  GT carbon in aboveground biomass, while the contribution of ECM and ERM plants constitutes 100 GT and 7 GT, respectively (for comparison, nonmycorrhizal plants contribute 29 GT carbon in terrestrial aboveground biomass) (Soudzilovskaia et al. 2019). Generally, there is an increasing trend in global AM coverage due to the increase of agricultural activity (Ellis et al. 2010, Soudzilovskaia et al. 2019).

Apart from morphological differences, different types of mycorrhizal fungi also exhibit distinct nutrient acquisition strategies. AMF, predominantly scavenge for inorganic soil nutrients and mostly provide plants with phosphorus and water, while ERMF and ECMF enable plant uptake of most micro- and macronutrients, including nitrogen in organic forms (Read and Perez‐Moreno 2003, Smith and Read 2008).

Together, this variability in forms and functionalities of mycorrhizal associations, enables a large spectrum of impacts of mycorrhizas on the functioning of soil (Tedersoo and Bahram 2019). Broadly, mycorrhizal impacts on soil processes could be summarized as ‘direct’ and ‘indirect’ effects (Rillig 2004). The ‘direct’ effects are associated with all aspects of the functioning of mycorrhizal fungi beyond nutrient provisioning to plants. The ‘indirect’ effects are associated with the mycorrhizal fungal contribution to plant nutrition, and therewith, the impacts on plant fitness affecting plant biomass and arguably plant ecophysiological traits (Cornelissen et al. 2001, Averill et al. 2019). The latter link, however, has been argued to be solely driven by taxonomical relatedness of ECM plant species (Koele et al. 2012). Among the multiple facets of mycorrhizal impacts on ecosystems, especially the ‘direct’ mechanisms of mycorrhizal impacts of soil processes (i.e. mechanisms through which mycorrhizal fungi govern soil biogeochemical cycles) remain poorly understood.

### Direct mycorrhizal fungal impacts on soil biogeochemical cycling

There is growing evidence that mycorrhizas affect soil biogeochemical cycles, with a magnitude of impact that is likely comparable to or even exceeds that of abiotic conditions (Heijden et al. 2015, Huang et al. 2022a). Yet, to date, the exact magnitude of these impacts remains unknown.

Contradicting evidence has been accumulated in regard to each of the aspects of distinct effects of mycorrhizal fungal guilds on soil processes (Table 1). The most striking contradictions and uncertainties are manifested across the following four domains: (i) impacts of individual mycorrhizal fungal guilds on soil carbon differ between the tropics and temperate zones (Fernandez and Kennedy 2016, Barceló et al. 2022), which suggests that key aspects of the mechanisms attributed solely to mycorrhizas’ activity might instead be driven by other aspects of ecosystem functioning, or by complex interactions between mycorrhizal fungal guilds and climatic conditions (Guo et al. 2024). (ii) Contributions of different mycorrhizal fungal guilds to carbon transfer and to processes occurring in distinct soil carbon pools (e.g. fresh plant litter, mycorrhizal fungal biomass, and soil organic matter at distinct depth levels) appear to differ (Cheeke et al. 2017, Frey 2019), while we are still far from understanding the full complexity of these phenomena. (iii) While many studies consider ECMF and ERMF fungal guilds as a joint pool (e.g. Averill et al. 2014, Ward et al. 2022), mechanisms by which they influence soil processes, such as their use of nutrient sources, nutrient transformation, and belowground carbon allocation and distribution, are likely to differ significantly (Lindahl et al. 2002, Ward et al. 2022, Hawkins et al. 2023). This possibly leads to conceptual failures in framing theories about the nature of impacts of distinct mycorrhizal guilds, specifically about the effects of ERMF on ecosystem functioning. Finally, very little is known about (iv) the contribution of distinct mycorrhizal fungal guilds to the formation of carbon pools with varying stability levels (particulate organic matter versus mineral-associated organic matter), although there is a growing evidence that these contributions also differ (Cotrufo et al. 2019, Lang et al. 2023).

**Table 1.** The current knowledge gaps in regard to impacts of main mycorrhizal fungal guilds, AMF, ECMF, and ERMF on soil processes.

**Mechanism 1 - mycorrhizal mycelial carbon pool**	
How much carbon from fresh photosynthate is allocated belowground to mycorrhizal fungi compared to how much is exuded into the soil directly through the root?	It is known that globally, ECM plants transfer approximately twice as much carbon to their mycorrhizal fungal partner as AM plants (2.47 versus 1.07 GT C per year globally) (Soudzilovskaia et al. 2015a, Hawkins et al. 2023). However, contrasting evidence exists regarding rhizosphere carbon fluxes in AM versus ECM plants, with observed variability across sites and mycorrhizal types (Keller et al. 2021). Despite these findings, the relative contribution of carbon from direct root exudates versus mycorrhizal fungal exudates to the rhizosphere remains unclear. Furthermore, the efficiency at which received carbon is utilized and transformed by distinct mycorrhizal fungal guilds, as well as the proportion directly exuded in the soil, remains poorly understood, particularly in relation to different mycorrhizal types.
What is the lifespan of AMF, ECMF, and ERMF?	Contradictions are found in reports on the overall lifespan of AMF and ECMF, with values ranging from 5 days (Staddon et al. 2003) to 5 months (Pepe et al. 2018) for AMF, and 120 days to and 831 days for ECMF (Fernandez et al. 2013). This considerable uncertainty can be attributed to the variations in methodological approaches (e.g. hyphae survival after plant shoot removal), which explain the survival of fungi without plants rather than the natural turnover rate of a hyphae. Additionally, phylogenetic differences may play a significant role in these discrepancies (Fernandez et al. 2016). Furthermore, no comparable data exists regarding the lifespan of different mycorrhizal fungal on intact hosts, nor the rate at which hyphae lose viability and are renewed under nonstress conditions.
What is the production rate and turnover rate of AM, ECM, and ERM extraradical fungal biomass?	
What are the decomposition rates of AM, ERM, and ECM extraradical fungal biomass?	While some data is available on the decomposition of roots colonized by AMF and ECMF (Langley and Hungate 2003), overall suggesting that ECMF colonization slows down the decomposition of roots more than AMF colonization (Langley et al. 2006), little is known about decomposition rates of extraradical mycelia (going beyond roots) of AMF and ERMF. To our knowledge, few studies have explored the differences between the chemical composition of AMF and ECMF (e.g. Huang et al. 2022). It has been established that molecules like melanin control the decomposition rate of mycorrhizal necromass (Fernandez and Koide 2014, Fernandez et. al. 2018); other components such as chitin and glucans have also been investigated (Mancinelli et al. 2023). However, our understanding of fungal necromass decomposition is limited to assessments of ECMF, lacking data about decomposition of AMF and ERMF hyphae. Therefore, the question of which chemical compounds, besides melanin, influence the rate of decomposition of these fungi remains open. Moreover, it is unclear which microorganisms are responsible for decomposition of the mycorrhizal fungal necromass. It is unlikely that the decomposition processes are similar among different types of mycorrhiza, as their chemical composition and associated microbiomes differ. It is known that microbial necromass contributes to more persistent soil organic matter pools (MAOM) and that the addition of microbial necromass in the soil can even decrease the overall decomposition rate of soil organic matter by the introduction addition of recalcitrant biomass components (Cheng et al. 2014). Considering this, we expect differences in contributions across mycorrhizal fungal guilds. However, the extent to which extraradical mycelium of specific mycorrhizal fungal guilds contribute to soil organic matter pools remains unclear.
Which microbial guilds/functional groups are primarily responsible for the decomposition of mycorrhizal necromass?	
Which compounds of mycorrhizal fungal biomass are persistent and intermittent to decomposition?	
Which soil organic matter pools does the mycorrhizal necromass contribute to?	
**Mechanism 2 - release of carbon components from roots**	
What are the decomposition rates of soil organic matter in environments dominated by AMF, ECMF, and ERMF?	The decomposition rate of various organic matter sources across different mycorrhizal environments remains largely unknown. To date, inconsistencies in the results obtained can be attributed to the context-dependent behavior of mycorrhizas (Fernandez and Kennedy 2016). Consequently, it is challenging to determine processes, such as soil organic matter decomposition and respiration rates in a comparable manner across the three mycorrhizal types.
What are the respiration rates of AMF, ECMF, and ERMF?	
Are there differences in underlying antagonistic mechanisms by which mycorrhizal fungi suppress saprotrophs in their environment, observed in the Gadgil effect?	The Gadgil effect has been extensively studied in ECM systems (Fernandez and Kennedy 2016), where the extent to which ECMF slow down the organic matter decomposition rate is thought to be driven by several underlying mechanisms (e.g. production of fungal/bacterial antagonists; Keller et al. 2005), nitrogen competition (Gadgil and Gadgil 1975). However, it remains unclear whether some of these mechanisms are species specific, or exist across the entire mycorrhizal guild, and whether they significantly differ between mycorrhizal guilds. These mechanisms have yet to be investigated in ERMF dominated environments. Given the enzymatic capacities of ERMF, a similar effect is believed to occur in ERMF systems (Ward et al. 2022). However, it remains to be determined whether other mechanisms observed in ECMF, or even unknown mechanisms, also are involved.
What is the magnitude of the Gadgil effect of ERMF?	
**Mechanism 2 - release of carbon components from roots**	
What are the mechanisms of priming imposed by AMF, ECMF, and ERMF?	Similar to the Gadgil effect, the priming effect is thought to vary among mycorrhizal fungal guilds, with the underlying mechanisms of priming being influenced by the environmental context (Cheng et al. 2014). This variability complicates assessments of how different types of mycorrhizal fungi contribute to or counteract microbial priming mechanisms, i.e. the production of fungal exudates and turnover of mycorrhizal biomass (Kuzyakov 2002). Currently, it is believed that there are no significant differences in the priming effect between AMF- and ECMF-dominated environments (Choreño-Parra & Treseder 2024, Hawkins et al. 2023), despite the likelihood of distinct mechanisms between the types. Additionally, no evidence exists concerning the contribution of ERMF to the priming effect, and quantifications of the magnitude of these mechanisms are virtually absent.
How is photosynthetically acquired carbon passed through the mycorrhiza into the soil to prime the environment?	It is well established that mycorrhizal fungi are able to exude labile carbon, which primes nearby saprotrophic organisms (Cao et al. 2022), and creates specific decomposition environments for bacteria (Odriozola et al. 2021). However, the extent to which carbon-rich molecules released by mycorrhiza are utilized by specific bacterial groups, and the precise functions for which this carbon is used, remains unclear.
What are the mechanisms of enhancing/slowing down decomposition rate due to activities of mycorrhizal fungi?	The presence of mycorrhizal fungi has been reported to enhance as well as slow down decomposition of individual pools of soil organic matter (Gagil versus priming effect). The interplay between these two phenomena is thought to be influenced by factors such as soil organic matter C:N ratio and climate conditions (Choreño-Parra and Treseder 2024). Due to this heterogeneity, the interaction between these two mechanisms remains unclear (Fernandez and Kennedy 2016), making it challenging to quantify the overall impact of mycorrhizal fungi on decomposition.
**Mechanism 3 - activity of mycorrhizal fungi mediates structure and composition of soil microbial communities**	
What are specific interguild interactions between ERMF, ECMF, and AMF?	Little is known about how mycorrhizal fungi of different types interact with fungi of other mycorrhizal types, and whether combinations of mycorrhizal fungal types have a synergistic, cumulative, or antagonistic effect on biogeochemical cycling (Fernández et al. 2022, Ward et al. 2022).
What are the guild-specific interactions of AMF, ECMF, and ERMF with microbial communities?	Although some progress has been made (Singavarapu et al. 2022), there is still little understanding of how mycorrhizal fungi interact with bacteria and saprotrophic fungi in their direct environment, or how they mediate the composition of microbial communities. Data on the impacts of ERMF is particularly scarce. Given that the ecophysiological characteristics of ERMF (e.g. enzyme production and exudation) are more similar to those of ECMF, it raises the question of whether this similarity extends to their interactions with bacteria and saprotrophic fungi.
**The effects of mycorrhizae on mineral weathering, soil acidity, and associated toxicity**	
How do different types of mycorrhizas alleviate environmental stressors, such as soil acidity and associated toxicity?	Significant differences in mineral weathering capacity and mechanisms (mostly enzyme production) have already been established between AMF and ECMF. ECMF exhibit a much higher capacity for mineral weathering compared to AMF (Taylor et al. 2009). ERMF that produce similar weathering agents to ECMF have been shown to have comparable weathering abilities (van Schöll et al. 2008). However, it remains unclear if these capacities are manifested across the entire guild or whether this is species specific. Extensive research has been conducted on the mycorrhizal fungal species *Serendipita indica* and its interactions with plants under environmental stressors such as drought, salinity, and nutrient deficiencies. Studies have shown that plants associated with *S. indica* exhibit enhanced tolerance to these stressors, with increased photosynthetic activity and upregulation of stress-responsive and antioxidant genes under adverse conditions (Varma et al. 2012). ERMF are prevalent in acidic soils and often encounter heavy metals, which makes them particularly interesting to investigate in these settings. However, while mechanisms of stress tolerance are likely similar to that of other mycorrhizal fungi such as *S. indica*, knowledge about their tolerance to acidity and metals is studied in limited species (Martino et al. 2000, 2002, Khouja et al. 2013), but general knowledge on their MyFE is lacking (Wei et al. 2022).
How do ERMF help ERM plants to cope with high acidity?	
How do ERMF contribute to the mineral weathering?	
What is the effect of elevated metal toxicity on AMF, ECMF, and ERMF?	

There are three main factors that exaggerate these knowledge gaps. First, plants featuring different mycorrhizal types have different growth forms: while AM plants are represented by all growth forms, the great majority of ECM plants are trees and shrubs, and the ERM plants are typically small to large shrubs (Brundrett and Tedersoo 2018, Soudzilovskaia et al. 2020). Consequently, experimental studies comparing ecosystem impacts of distinct mycorrhizal types are typically conducted with trees (e.g. Ferlian et al. 2018, Phillips et al. 2013), and are either limited to planted tree seedlings (and have to account for the fact that the build-up and activities of mycorrhizal fungal communities associated with seedlings do not fully represent those associated with mature trees; Hart et al. 2014), or are conducted in long-established vegetation stands developed on inherently different soils, which do not allow for a conclusive disentanglement of the effects of mycorrhizas and inherent effects of soil properties. Second, natural ecosystems rarely represent one single mycorrhizal type. Rather, we deal with a certain level of dominance of plants featuring one mycorrhizal type (for instance 80% of plant biomass is comprised by AM plants), and additional impacts of other mycorrhizal types (for instance 10% of plant biomass is ECM plants and 10% is ERM plants). Considering such communities as ‘purely AM’ is too simplistic, and estimating the additional impacts of ECMF and ERMF based on the aboveground biomass of ECM and ERM plants is impossible. Finally, most information regarding the effect of mycorrhiza on biogeochemical cycling has been obtained for AM and ECM. Knowledge on the impacts of ERM plants on soil processes is extremely scarce, despite the fact that ERM plants play important roles in a number of natural ecosystems, such as tundra, boreal forests, heathlands, and Mediterranean and South African shrublands (Tedersoo 2017).

By enabling an interface for direct nutrient exchange between plants and soil, mycorrhizas affect individual aspects of soil biogeochemical cycles through a complex set of partially interlinked mechanisms. Below, we address what is known about these direct mycorrhizal fungal impacts, and what remains disputable, and introduce a new framework of mycorrhizal fungal environments (MyFE) that can be used as a tool to streamline the identification of current knowledge gaps and increase our understanding of mycorrhizal impacts on soil processes.

#### Carbon and nutrient cycles

**Figure 1.**
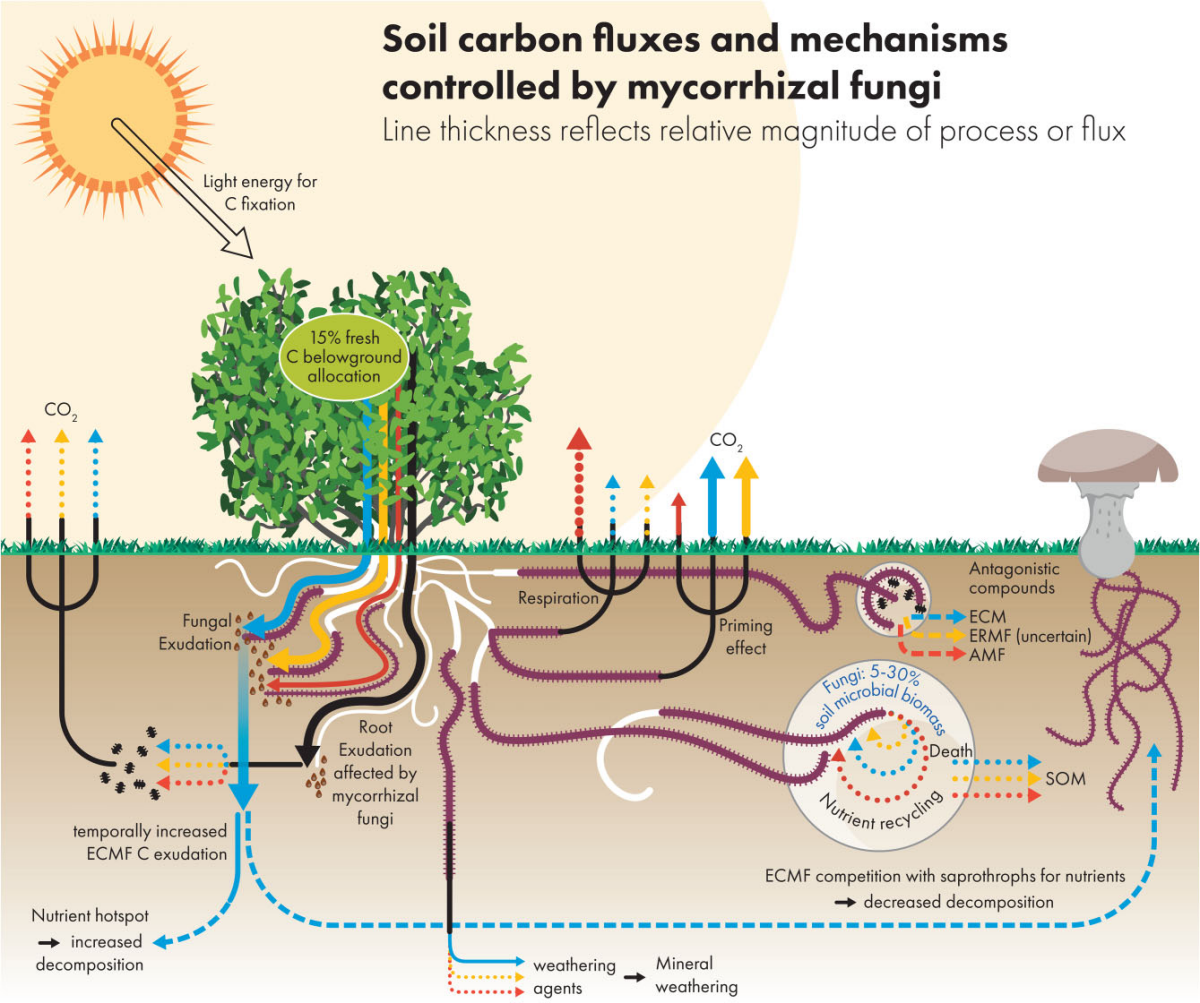
A schematic overview of the flow of carbon from atmosphere to soil according to ECM (blue), ERM (green), AM (red), and dotted lines indicate uncertainties.

There are three major pathways of direct mycorrhizal fungal impacts on soil carbon and nutrient cycles (Soudzilovskaia et al. 2015b, Frey 2019) (Fig. 1): (i) forming a carbon pool in mycorrhizal mycelium; (ii) affecting the release of carbon components from roots through root exudation; and (iii) mediating community composition and activity of saprotrophic organisms that facilitate or impede soil organic matter decomposition.

##### Mycorrhizal mycelial carbon pool

Plant allocation of photosynthetically fixed carbon into a network of mycorrhizal fungal mycelium constitutes the channel of direct transmission of carbon into the soil. Depending on mycorrhizal type and environment, mycorrhizas account for 5%–30% of the microbial biomass in soils (Leake et al. 2004), which in itself constitutes a considerable soil carbon pool. The build-up process of the mycorrhizal mycelial carbon pool in the soil is regulated through three processes, with the magnitude having been shown to differ between mycorrhizal types. These processes are: (i) the flux of the fresh photosynthetically fixed carbon from plants to mycorrhizal fungal partners, (ii) the life span of fungi in soil, and (iii) the process of decomposition of dead mycelium of mycorrhizal fungi.

The flux of fresh photosynthetically fixed carbon from plants to mycorrhizal fungal partners is greatest for ECM, with the AM fungal network receiving a comparatively lower fraction of plant carbon (Soudzilovskaia et al. 2015b, Hawkins et al. 2023). Net primary production allocation belowground had been estimated at 6.20% for AM, 13.10% for ECM, and 3.50% for ERM. On a global scale, the magnitude of this flux has been estimated at 1.07 GT C per year for AM symbiosis, ECM 2.47 GT C per year for ECM, and 0.03 GT C per year for ERM (Hawkins et al. 2023).

The next parameter shaping the mycorrhizal fungal carbon pools in ecosystems is the lifespan of mycorrhizal fungi. Little is known about it, with a handful of estimations available till now, suggesting that AMF have a considerably lower lifespan compared to ECMF, and virtually no data is available for ERMF. It has been reported that extraradical mycelium of AMF species can survive 5–6 days after severing the mycelium (Staddon et al. 2003) in sterile conditions, while in natural environments, this is likely to be accelerated due to the presence of mycelia grazers and damage caused by environmental stressors. However, recently it has been demonstrated that depending on the fungal species and distance from hyphae to the root, AMF could last up to 5 months, even if host plant shoots have been removed, thus suggesting that the survival of the extraradical mycelium of AMF is highly variable (Pepe et al. 2018). These reports, however, describe the survival of obligatory biotrophic AMF after being severed from their plant host, which does not accurately reflect the true lifespan and turnover rate of unsevered AMF in standard environments. For both AMF, and ECMF the lifespan is likely species specific. While many ECMF have a life span of ca. 120 days, the species *Cenococcum geophilum* can have a lifespan of 831 days (Fernandez et al. 2013). Moreover, the lifespan of ECMF may also be influenced by factors, such as soil nutrient availability, and the specific morphotype. The addition of nutrients such as nitrogen to soil has been shown to increase the lifespan of ECM, with the effect varying depending on the morphotype (Kou et al. 2017).

The decomposition rate of biomass of distinct guilds of mycorrhizal fungi also likely differs. Till now, only data on the decomposition rates of ECMF are available, which suggests that, despite the considerable interspecific variation (Brundrett and Tedersoo 2018), on average 80% of fungal necromass is lost within 2–8 weeks (Ryan et al. 2020). Recent research has demonstrated that the chemical composition of AMF and ECMF fundamentally differs in the aspects controlling organic matter decomposability (Huang et al. 2022). Yet, further research on the chemical composition of fungi from distinct mycorrhizal guilds, particularly ERMF, is needed.

Little is known about fungal mycelial traits underpinning fungal decomposition rates. The ratio of melanin: nitrogen has been shown to be a key factor controlling decomposition of ECMF and ERMF (Koide and Malcolm 2009, See et al. 2021, Fernandez and Koide 2014), with melanin being the most recalcitrant fungal tissue component, and nitrogen concentrations being positively correlated to fungal decomposability (Berg 2000, Koide and Malcolm 2009, Fernandez and Kennedy 2018).

##### Release of carbon components from roots

Mycorrhizal fungi influence soil carbon pools by modulating processes of root exudation and through the release of fungal exudates (Keller et al. 2021). Distinct mycorrhizal types can therefore directly affect the rhizosphere environments of plants, which makes mycorrhizas key determinants of soil rhizosphere processes (Leake et al. 2004, Lin et al. 2017, Keller et al. 2021, Tedersoo et al. 2021). There are two active pathways:

First, mycorrhizal fungi exert, to some extent, control over the root exudates released into the rhizosphere, as they form an interface between soil and root. As carbon is allocated belowground by the plant, distinct mycorrhizal guilds are believed to have different impacts on the amount carbon allocated belowground and entering the soil (Keller et al. 2021, Hawkins et al. 2023). Additionally, mycorrhizal fungi may increase the belowground allocation of carbon. It has been shown that plants inoculated with ECMF accumulate more photosynthates in the roots in comparison with nonmycorrhizal plants (Wu et al. 2002). Exudates not used by the mycorrhizal fungi become available to the other soil microorganisms associated with mycorrhizal hyphae (Kaiser et al. 2015). It has been reported that root derived carbon inputs may result in an increase, decrease, or no net change in soil organic matter (Cheng et al. 2014, Sokol and Bradford 2019).

It has been suggested that when mycorrhizal fungi approach nutrient rich spots, plant hosts increase the labile carbon transport to the fungus to stimulate decomposition of organic matter by the hyphae associated saprotrophic microorganisms in the soil, making more nutrients available (Farrar et al. 2003, Badri and Vivanco 2009, Kaiser et al. 2015). However, the mechanisms controlling this phenomenon, such as the degree of efficiency at which labile carbon is used and transformed by mycorrhiza, and how this differs between mycorrhizal types, remains poorly understood.

Second, AMF, ECMF, and ERMF themselves secrete many carbon-rich compounds (Keller et al. 2021). For ECMF and ERMF, these constitute components such as oxalate and chelators, which cause the liberation of micronutrients through mineral weathering, increasing their availability for plant uptake (Landeweert et al. 2001, Phillips et al. 2013). These carbon-rich compounds, such as oxalate, exuded by the fungi can further be used as a carbon source by bacteria in the rhizosphere (Sun et al. 2019). AMF can produce glomalin-related proteins, which changes the soil properties in their direct environment, promoting soil aggregation, and contributing to soil carbon storage (Singh et al. 2013). Moreover, AMF-driven glomalin supply in the soil is correlated to the amount of photosynthate allocated to the plant (Taylor et al. 2009).

Fungi of distinct mycorrhizal types have different extracellular enzymatic properties, which also alter their direct soil environment, and soil carbon. Ectomycorrhizal and ERM fungi can produce hydrolytic and oxidative extracellular enzymes, such as lignases, cellulases, and polyphenol oxidases (Lindahl and Tunlid 2015), that decompose organic matter (Tunlid et al. 2022) and contribute to the degradation of plant material (Read and Perez‐Moreno 2003). AMF lack enzymes that are capable of breaking down complex organic matter in their environment, but they may produce enzymes, such as acid phosphatase for nutrient acquisition purposes (Sato et al. 2015). Besides the components related to nutrient uptake, mycorrhizal fungi may release a large group of secondary compounds, such as metabolites, that have a hormonal, excretory, or antibiotic role (Keller et al. 2005), and at the same time constitute a contribution to soil carbon pools.

The ultimate suits of compounds released into the soil by mycorrhizal fungi differ between AMF, ECMF, and ERMF. For enzymes, these differences between mycorrhizal types are relatively well understood, and are related to the capacity of fungal enzymes to break down soil organic matter (Tedersoo and Bahram 2019). AMF lack decomposer capacities and therefore take up more mobile inorganic nutrient forms, hence their preferred uptake of inorganic nitrogen (Chowdhury et al. 2022) and phosphorus (Riaz et al. 2023). Since ECM and ERM have more extensive saprotrophic capacities, they have the ability to break down more complex materials, enabling them to mine nutrients from more recalcitrant sources, and to take up nutrients in their organic form.

##### Activity of mycorrhizal fungi mediates soil microbial communities

Within the mycorrhizosphere, the microenvironment surrounding mycorrhizal roots, mycorrhizal fungi actively mediate the composition and activity of soil microbial communities. Therefore, in association with bacteria, mycorrhizal fungi form intricate holobiont interactions with their plant hosts (Mogge et al. 2000, Frey-Klett et al. 2007). Moreover, mycorrhizal hyphae harbour a highly diverse microbial community, including bacteria that colonize both fungal hyphal surfaces and endofungal locations, where they play essential roles in mycorrhizal establishment and maintenance (Mogge et al. 2000, Frey-Klett et al. 2007).

Within the mycorrhizosphere, the enzymatic activity of mycorrhizal fungi facilitates the decomposition of complex organic molecules, enabling nutrient uptake. These decomposition products attract other microorganisms for further degradation and subsequent processing (Talbot et al. 2008). The release of decomposition products, may alter the pH of mycorrhizosphere, which, in turn influences the bacterial community composition (Kielak et al. 2016, Johnston et al. 2019). Additionally, carbon-rich exudates released by mycorrhizal fungi also shapes microbial populations (Miransari 2011, Itoo and Reshi 2013). Oxalate, produced by ECMF, drives shifts in functional groups of bacteria, attracting specific functional groups of bacteria that stand in for oxalate degradation, as well as increasing the abundance of nitrogen-fixing bacteria (Sun et al. 2019). Through these interactions, mycorrhizal fungi actively shape their associated microbial communities, forming associative networks with bacteria, and ultimately influencing processes such as plant litter decomposition (Odriozola et al. 2021).

By taking up nutrients from soils, mycorrhizal fungi also compete for nutrients with saprotrophic microorganisms. The best-known phenomenon related to this mechanism, the Gadgil effect (Gadgil and Gadgil 1971), is characterized by a reduced rate of soil organic matter decomposition in the presence of ECMF, due to competition for nitrogen with saprotrophic microorganisms, leading to increased carbon sequestration. By taking up nitrogen selectively and more efficiently than saprotrophic organisms, mycorrhizal fungi increase the carbon to nitrogen ratio of soil organic matter, leading to increased carbon sequestration (Gadgil & Gadgil 1971). Furthermore, ECMF possess competitive mechanisms, including the production of antagonistic chemical compounds, such as volatile organic compounds, antimicrobial, and antifungal compounds that suppress and limit the activity of other saprotrophic microorganisms (Krywolap and Casida Jr. 1964, Garrido et al. 1982, Kope and Fortin 1990). Also, being less limited in carbon in comparison to their saprotrophic counterparts, ECMF are capable of allocating more resources to produce these antagonistic compounds (Keller et al. 2005). Finally, ECMF may also tap into the biomass of living saprotrophs using those as a source of nutrients, and therewith suppressing the decomposition of litter (Cairney and Meharg 2002). However, due to the complexity of the soil organic matter decomposition process, a lot of inconsistent results have been obtained around this topic, where in some cases the presence of ECMF did not lower the decomposition rate but accelerated it (Brzostek et al. 2015). This occurs when root exudates or carbon-rich compounds released by mycorrhizas increase the microbial activity, leading to an increased decomposition rate of soil organic matter, known as the priming effect (Dalenberg and Jager 1989, Kuzyakov et al. 2000). These shifts between the priming effect and the Gadgil effect can be attributed to the context-dependent characteristics of mycorrhizal fungi, where different outcomes are observed depending on the biotic and abiotic conditions (Cheng et al. 2014, Fernandez and Kennedy 2016).

Beyond biochemical interactions, mycorrhizal fungi can also physically impact microbial activity. Mycelial networks have been shown to bridge soil air gaps, facilitating bacterial movement and access to new microhabitats (Nazir et al. 2014). The diversity of ECMF and their distinct exploration strategies (Agerer 2001) suggest that different species differentially influence bacterial distribution. Furthermore, the composition of microbial communities may vary depending on hyphal morphology and type (Voronina et al. 2011).

##### Mycorrhizal impacts on mineral weathering and micronutrient availability

Mineral weathering plays an important role in several soil processes including plant nutrition, podsolization, and soil horizon formation (Van Breemen et al. 2000). Additionally, mineral weathering mediates the effects of soil acidification by freeing bioavailable elements that act as a buffer, influencing the plant’s ability to overcome natural stresses. ECMF can increase the micronutrient availability in soils, as they are able to exude weathering agents, such as oxalate, that are capable of breaking down minerals. This mineral weathering allows mineral phosphorus and other micronutrients, such as calcium and magnesium, to become accessible for plant uptake, thereby increasing soil fertility (van Schöll et al. 2008). The scale of micronutrient mining is specific to the species of mycorrhizal fungi (van Schöll et al. 2008). Although this phenomenon has been observed in ECMF, the capacities for mineral weathering remains unknown for ERMF. AMF are believed not to excrete mineral weathering agents, such as organic acids and chelators, and therefore, their contribution to mineral weathering is considered to be less effective than that of ECMF and possibly ERMF. However, phenomena, such as tunnelling, i.e. the formation of hyphae-shaped microscopic tunnel-like structures on mineral substrates (Smits 2006), observed during mineral weathering can also be found in AM forests, where ECMF are absent. This suggests that the excretion of organic acids of AMF may either be overlooked, due to the presence of saprotrophic microorganisms in their environment, or a result of combined acidification attributed to the release of biotic agents in the rhizosphere (Koele et al. 2014).

#### The effects of mycorrhizas on soil acidity and associated toxicity

Mycorrhizal fungi affect soil acidity in a number of ways, by producing and releasing organic acids, by interactions with bacteria and other microorganisms, and by the process of mineral weathering itself (Finlay 1995). Soil acidification increases the solubility of iron and aluminum (Al), causing their leaching from the soil, which strongly affects plant nutrient uptake. Moreover, high levels of soluble Al negatively impact plant growth and physiology. Even though soil acidification may negatively influence plant and mycorrhizal fungi interactions by influencing the allocation of carbon to the mycorrhizal fungi and affecting the uptake of other minerals, such as magnesium and calcium (Finlay 1995, van Schöll et al. 2008), mycorrhizal infection helps plants mitigate these adverse conditions (Finlay 1995).

Both ECMF and AMF increase plant access to nutrients, therewith mitigating the toxicity of acidic environments. Seedlings colonized with ECMF obtain a relatively higher nutrition than nonmycorrhizal seedlings in elevated metal conditions (Ahonen-Jonnarth et al. 2003). Hyphae on the root tip block the main binding sites for Al, diminishing its uptake. Moreover, Al is accumulated in mycelium, and organic acids, which act as a chelating agent, are produced so that Al remains sequestered internally or externally (Eldhuset et al. 2007, Machuca et al. 2007).

AMF likewise, are able to detoxify Al in the rhizosphere by immobilizing it in fungal cell vacuoles or binding it into the cell wall. Arbuscular mycorrhizal fungal associations may even increase the release of root exudates, which bind to Al limiting its toxic effect (Seguel et al. 2013). However, similar to ECMF, the effects of AMF on Al toxicity vary between species of AMF (Seguel et al. 2013).

## MyFE- new framework embracing mycorrhizal fungal impacts on soil processes

There exists a unanimous consensus that mycorrhizal fungi strongly affect fundamental soil processes, and it has been suggested that soil processes are to a large extent determined by the mycorrhizal types dominating in an ecosystem, with AMF and ECMF imposing contrasting impacts on the majority of soil processes (Read and Perez‐Moreno 2003, Leake et al. 2004, Phillips et al. 2013, Soudzilovskaia et al. 2015b).

**Figure 2.**
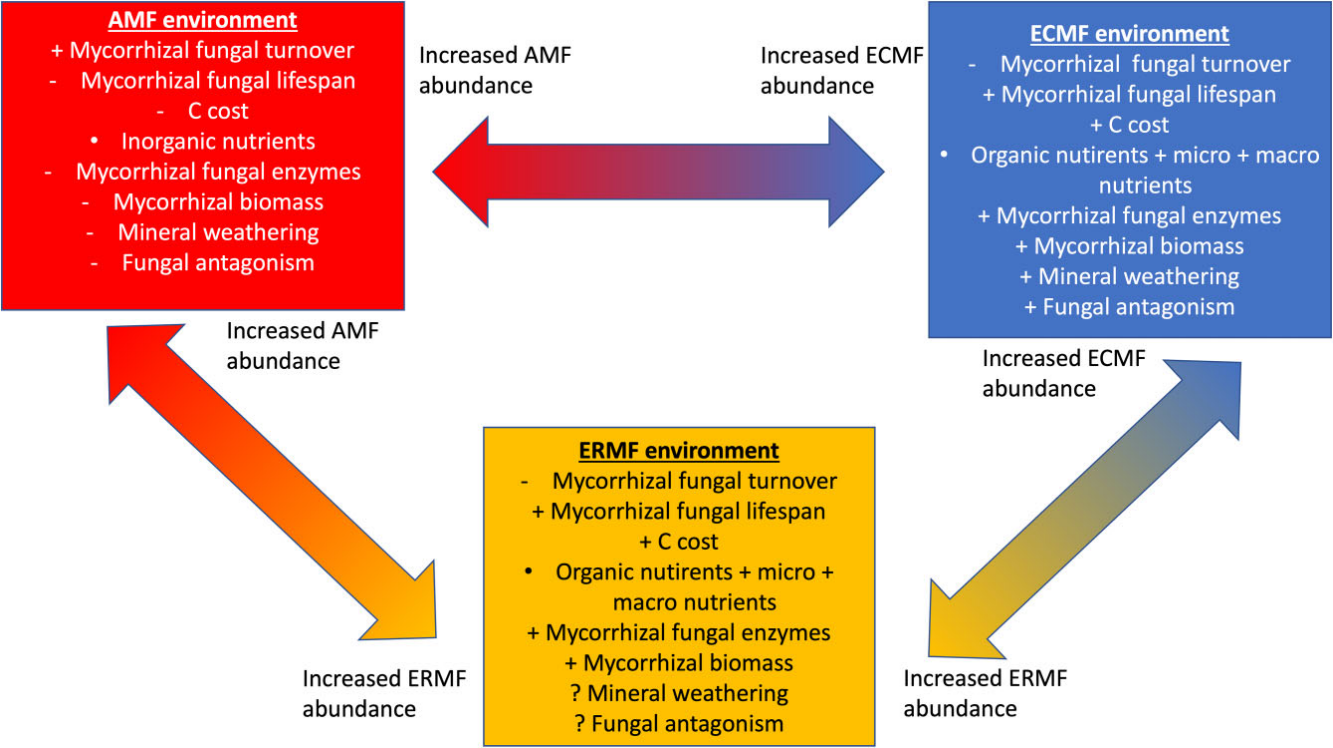
Differences among mycorrhizal fungal guilds in the direct mechanisms by which they influence soil carbon, thereby significantly altering their immediate environment and shaping their MyFEs. The relative magnitude of these impacts remains poorly understood.

To advance the understanding of mechanisms through which mycorrhizas impact soil functioning, and to conceptualize their contribution to soil biodiversity and biochemical properties, we propose a framework of MyFE. The MyFE represents unique soil environments created by presence and activity of each individual mycorrhizal fungal guild. This involves processes that directly affect carbon and nutrient cycles such as lifespan and turnover rate, enzyme expression, and carbon exudation, and attraction of microorganisms, and possibly includes other mechanisms that are currently overlooked or understood inadequately. These unique features will therefore shape soil biogeochemical cycles and biodiversity into AMF, ECMF, or ERMF-typical soil environments, (Fig. 2).

The newly introduced MyFE concept builds upon the concept of the mycorrhizosphere, the immediate soil environment influenced by mycorrhizal fungal hyphae through altered exudation and microbial recruitment. MyFE offers a broader, more dynamic perspective, considering the combined effects of different mycorrhizal types. Rather than focusing solely on local exudation and microbial interactions, MyFE extends beyond the rhizosphere, hyphosphere, and extraradical hyphae into bulk soil. MyFE, therefore uncovers emergent antagonistic and synergistic effects across soil compartments that would otherwise be missed or exaggerated in smaller-scale observations. Ultimately, this framework provides a systems-level understanding of mycorrhizal influences on ecosystem functioning, adding to the conceptual limitations of the traditional mycorrhizosphere.

The MyFE framework therefore provides a comprehensive approach to better understand the impacts of mycorrhizal fungal guilds on soil processes. By integrating individual processes associated with these guilds, the MyFE concept allows for the characterization of soils based on the presence and activity of mycorrhizas, where the abundance of specific guilds leads to distinctive soil properties.

This framework offers a novel perspective on soil carbon and nutrient dynamics by linking mycorrhizal activity, abundance, and diversity to specific soil processes and functions. By identifying critical knowledge gaps and facilitating more targeted investigation, this framework has the potential to significantly increase our understanding on mycorrhizal abundance and diversity and their contribution to specific soil properties and functions. Therefore, embracing this concept enhances the ability to predict soil process and assess soil health, making it highly applicable in several domains.

First, research on mechanisms within MyFE can be integrated in advanced ecological computational models that focus on below- and aboveground carbon fluxes and carbon sequestration, based on vegetation type and mycorrhizal fungal guilds (Huang et al. 2022a). This enables predictions about how different mycorrhizal communities might influence soil carbon and nutrient dynamics and ecosystem functions at both local and global scales. For example, shifts from AM-dominated to ECM-dominated to ERM-dominated systems can be modelled to predict changes in soil carbon and nutrient fluxes, as well as soil carbon storage. By addressing knowledge gaps concerning the impacts of different mycorrhizal guilds on soil functions, we can improve predictions concerning their effects on biogeochemical cycling. In the context of climate change, MyFE can also help to predict and monitor future carbon fluxes, assess carbon sequestration, and storage potential under changing environmental conditions.

Second, adopting MyFE could also have significant implications for land use management and agricultural practices. It can advance our understanding of crop carbon use efficiency, inform soil amendments, improve soil health monitoring, indicate local soil carbon dynamics, and guide sustainable land management, bioremediation, polluted soil management, and urban planning.

Ultimately, by defining the processes through which different mycorrhizal fungal guilds shape soil parameters, researchers can use the MyFE framework to interpret results from observational studies and contextualize outcomes of controlled lab experiments. The framework also highlights the distinct roles of ERMF and ECMF, which are often not separated in ecological analyses (Averill et al. 2014, Ward et al. 2022). This distinction is crucial for obtaining knowledge that is currently scarce, and identifying the similarities and differences between these distinct mycorrhizal fungal guilds (Ward et al. 2023).

Embracing this concept allows streamlined progress in research of mycorrhizal ecology, by recognizing the existence of differential impacts of mycorrhizal fungal guilds on soil, and allowing a better understanding of how MyFE’s are shaped. Importantly, MyFE-driven processes may have opposing directions, and may partially compensate for one another. For instance, while ECMF constitute larger standing biomass in soil than AMF, and therewith positively contribute to soil carbon (Soudzilovskaia et al. 2015b), root exudates of ECM plants contribute less to soil carbon than root exudates of AM plants (Keller et al. 2021). Embracing the MyFE framework, we elaborate the knowledge gaps in regard to the impacts of mycorrhizal guilds on soil processes, summarized in Table 1, and visualized in Fig. 1.

## The way forward

The following experimental framework, Mycotron, principally enables the quantitative testing of the concept of MyFE. To alleviate the confounding effects of soil types and history, an experimental setup should constitute a common garden built on a uniform soil type, using plant species featuring different mycorrhizal guilds. To compare the impacts on ecosystem functioning among all three prominent mycorrhizal types (AM, ECM, and ERM), plant hosts of each types should be included. To further eliminate confounding factors related to plant species selection, the experiment should employ adult plants of the same growth form and with similar ecophysiological traits. Finally, to quantify the impacts of specific mycorrhizal types, gradients of mycorrhizal dominance should be provided.

## Mycotron- mycorrhizal diversity-gradient experiment

**Figure 3.**
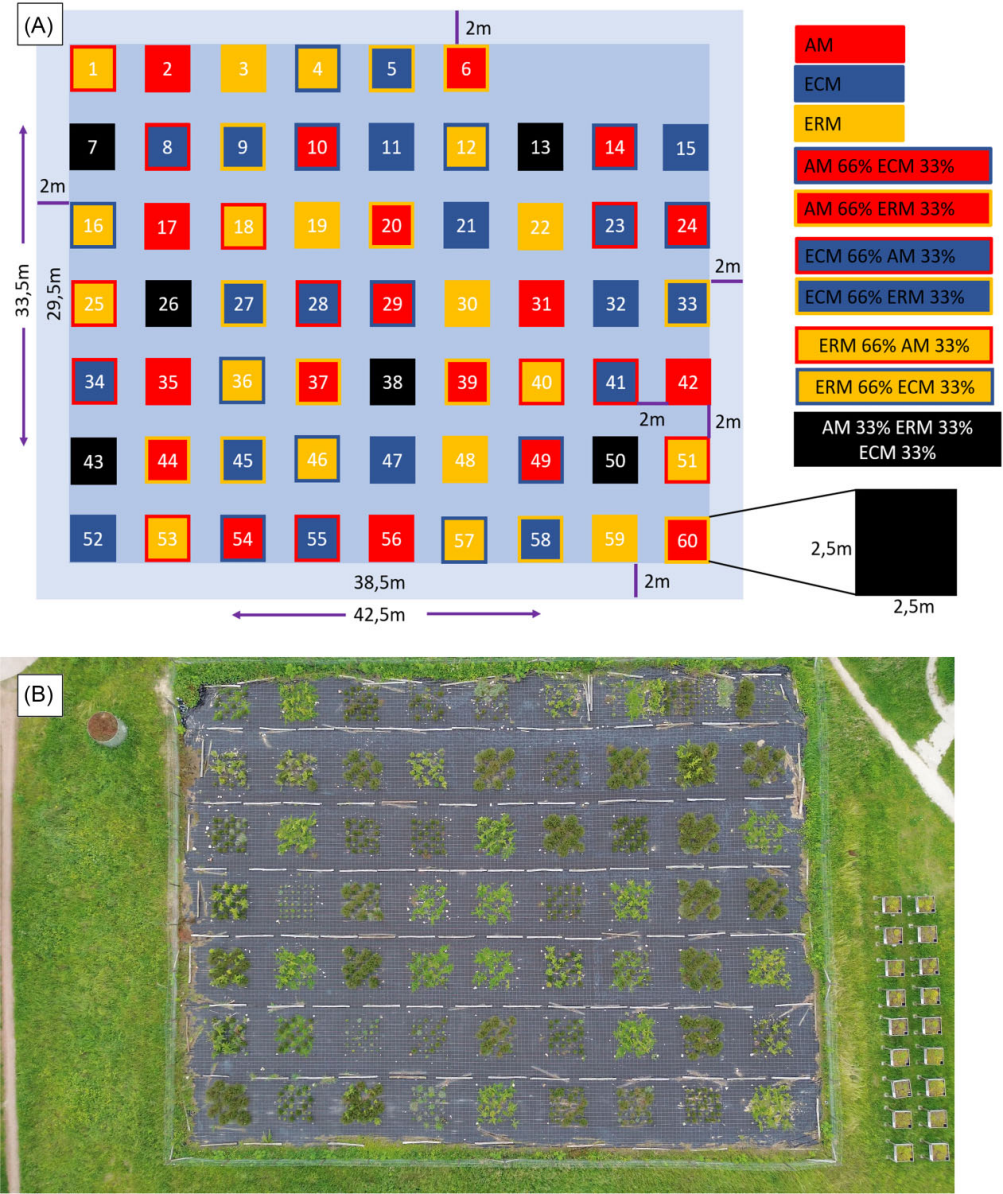
(a) Schematic overview of the experimental plots. The experimental design entails 10 distinct mycorrhizal conditions, each replicated six times. (b) Air photograph of the experimental site.

As a proof of concept, we established a long-term experimental field at National Park Hoge Kempen (NPHK). Sixty subplots of 2.5 m × 2.5 m with a margin of 2 m in between were established on sandy soil (Fig. 3). The entire study site has the size of 33.5 m by 42.5 m. We aimed to enable comparison of the three most abundant mycorrhizal types, ERM, ECM, and AM for soil impacts. We selected three plant species per each of the three mycorrhizal types (Table 2). Plant species were chosen to differ as little as possible in ecophysiological traits, besides the mycorrhiza type. We have opted for a shrub growth form, because it is the only form truly shared between all three mycorrhizal guilds. These similar traits in plants allow for the plant effect to be minimized.

**Table 2.** The plant species used in the experiment and their respective mycorrhizal types.

Mycorrhizal type	Plant species
AM	*Juniperus communis*
	*Cotoneaster dammeri*
	*Hypericum calycinum*
ECM	*Dryas octopetala*
	*Helianthemum nummularium*
	*Halimium umbellatum*
ERM	*Calluna vulgaris*
	*Erica cinerea* ‘Pallas’
	*Vaccinium vitis-idaea*

**Figure 4.**
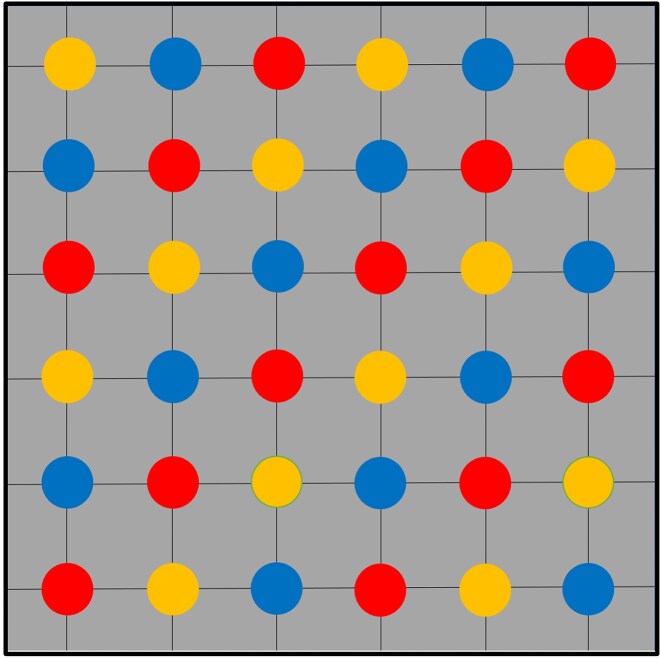
A schematic overview of plant locations in Mycotron experimental plots. Each plot holds a total amount of 36 plant individual. Plants are planted 40 cm apart from each other. Plot margins are 25 cm. Different colours can be attributed to different plant species, species 1, species 2, and species 3.

On each plot, 36 plant individuals featuring developed mycorrhiza were planted with 40 cm spacing to leave sufficient space for growth and implementing tools for future experimentation (Fig. 4). All the plants were planted bare rooted.

Different plant species were combined in different proportions to establish a gradient of mycorrhizal dominance, spanning 0%–33%–66%–100% dominance of each mycorrhizal type (Fig. 3). In this manner, the following conditions were created: pure mycorrhizal types (100% ERM, 100% ECM, and 100% AM), dual mixtures with one dominantly present (66%/33% ratio), and plots with all types combined evenly (33%/33%/33%), each condition occurring six times throughout the experiment (Appendix 1).

This type of experiment can be used to acquire knowledge that is currently lacking in the field (Table 1). The knowledge gaps presented in Table 1 focus on the ecological mechanisms driven by functioning of mycorrhizal guilds and their inherent differences and similarities. Mycotron introduces a method to quantitatively assess these mechanisms, eliminating the confounding effects such as impacts of differences between plant functional types and soils, and serves as a tool to bridge highly controlled lab studies, and observational field studies.

The Mycotron experiment also has important limitations. First, observed processes may be specific to soil parameters of the experiment (e.g. sandy soil). Therefore, fungal diversity and fungal species composition within the mycorrhizal guilds may be affected by a cross-guild environmental selection for fungal species that share similar environmental adaptations. While this limitation is unavoidable within a local experimental setup, it is partly mitigated by the fact that specific soil conditions promote physiological adaptations that will likely be similar within mycorrhizal guilds. Consequently, such adaptations are expected to manifest consistently across all mycorrhizal guilds, thereby mitigating the risk of exaggerating differences among guilds. Besides, given the huge differences in ecophysiology and biomass chemical composition between mycorrhizal guilds rather than between fungal species within guilds (Huang et al. 2022), the measurements conducted in Mycotron will still be informative about guild-specific MyFE processes. Yet, the next necessary step is to enable the understanding of the role of variation in soil types and climate on the expression of MyFE-associated mechanisms in soil.

Herewith, we accept an additivity assumption for the impacts of mycorrhizal types and other environmental factors (soil and climate). The experimental evidence accumulated thus far, does not contradict this assumption, but further investigations across distinct environmental conditions are needed to fully validate it. Results obtained from this experiment have the potential to be compared to the outcomes obtained in similar experimental set-ups with different vegetation types (Ferlian et al. 2018). Moreover, with this proposed framework of MyFEs, we encourage other soil researches to initiate their own common garden experiments that focus on mycorrhizal fungal guilds to create gradients of MyFEs across a wide range of soil and climate environments.

A second, important limitation deals with the fact that such controlled experiment allows only a limited plant species number to be included into the experimental setup. The plants used in the experiment cannot represent the entire species diversity of each mycorrhizal type. Besides, diversity of plant species and growth forms differs between mycorrhizal guilds, making a unified approach impossible. To our knowledge, this limitation is true to all similar experiments (e.g. tree seedlings), and can only be overcome by comparing to complementary experiments (Ferlian et al. 2018).

## MyFE-associated research questions to be answered with Mycotron

Our overview of current knowledge gaps in regard to the functioning of mycorrhizal fungi highlights the large uncertainties related to direct (sensu; Rillig 2004) contribution of fungi of distinct mycorrhizal guilds to biochemical cycles. The proposed framework of MyFE, allowed us to identify critical aspects that need to be covered in experimental assessments of mechanisms of mycorrhizal fungal impacts on soil processes, and yielded a set of criteria which we strive to fulfil in the design of the Mycotron experiment. While this experiment could not cover a complete set of the knowledge gaps identified in this paper (Table 1), it provides a comprehensive array of possible analyses, and experimental set-ups aimed to solve a large set of urgent research questions concerning mycorrhizal impacts on soil carbon and nutrient cycling, as well as on soil ecosystem responses to abiotic stresses. Below, we discuss the types of assessments and research questions concerning the quantification of the MyFE concept, possible to address in this experiment.

### Transfer of carbon from plant to soil via mycorrhizal fungi

In the first years after establishment, the Mycotron experiment allows direct comparative analysis of the turnover rate and lifespan of AMF, ECMF, and ERMF. All plants have the same age, and are initially planted into the same soil at the same point in time. Therefore, in the beginning, when soil has not yet been significantly affected by fungal activities, all fungi will be subjected to very similar abiotic conditions, eliminating the confounding impacts of differences in soil properties. The use of low state plants (shrubs) in the experiment allows isotopic labelling of individual plants, to trace carbon transfer form plants to fungi, in a standardized manner. This provides the opportunity to determine the carbon flux integrated into the biomass of fungi of different mycorrhizal types. Subsequently, the life span of individual fungal species, and their succession could be assessed as well.

Further, the isotopic labelling technique allows examining root exudation of plants that belong to distinct mycorrhizal guilds. This allows assessments of the fractionation of carbon flow between mycorrhizal fungi and root exudates, and determining the carbon costs and carbon efficiency of different mycorrhizal fungal types, independently from soil conditions.

### Processes of organic matter decomposition and incorporation of carbon into mineral associated organic matter

Understanding how the dominance of fungi from distinct mycorrhizal types affect soil organic matter decomposition, relative to abiotic soil parameters, remains one of the most critical and unresolved challenges in mycorrhizal research. The Mycotron experiment creates an ideal set up for the execution of various litter transplantation experiments of e.g. plant leaf, plant root, and fungal litter, among different mycorrhizal environments and mycorrhizal mixtures, that will provide insights into the impacts of mycorrhizal fungal types on soil organic matter decomposition processes. Further, soil trenching can easily be implemented on the plots to control the access of mycorrhizal fungi to litter transplants, adding another level of control, and allowing assessment of mechanisms associated with the Gadgil effect (Fernandez and Kennedy 2016). Finally, initially equal soil conditions allow the assessment of the mechanisms that form minerally associated organic matter in the context of MEMS theory (Cotrufo et al. 2013, 2015). Hereto, methods similar to that proposed by Sokol and Bradford (2019) could be applied.

### Mycorrhiza mediation of the soil microbiome and soil animal communities

To assess bacterial, fungal, and soil animal communities associated with different types of mycorrhiza, microbiome and soil invertebrate community analyses can be conducted on soil samples collected at the Mycotron experimental plots. Interactions between fungi and bacteria in the rhizosphere can be assessed with carbon-tracing methods of amino sugars (Klink et al. 2022). Additionally, due to the identical starting conditions of the experiment, temporal dynamics and cumulative effects of these interactions can be elucidated, providing insights into how this relationship evolves over time.

### Mineral weathering, acidity, and metal toxicity

By the manual addition of minerals, mineral weathering processes, such as tunnelling in rocks and the release of weathering agents, can be investigated in the Mycotron experiment. By isotopically labelling individual plants, the mycorrhizal origin of organic acids responsible for mineral weathering can be recalled. Therefore, the role of AMF and ERMF in mineral weathering, could be investigated more in-depth.

Environmental stressors, such as drought or metal toxicity, can also be simulated on the experimental plots, and the physiological responses (e.g. changes in gene expression, mycorrhiza morphology, plant yield) of mycorrhizal fungi can be investigated accordingly.

### Exclusive assessments of the role of ericoid mycorrhiza in soil ecosystem functioning

Till now, the great majority of assessments of mycorrhizal fungal impact on soil processes have been limited to comparisons of AMF- and ECMF-dominated systems, with ecosystems dominated by ECMF often including some ERM vegetation, which is often common in forests dominated by ECM trees. Besides the rare occurrence of a purely ERM-dominated ecosystem, the predominant shrub life form of ericaceous plants presents another obstacle in comparing the impacts of ERMF on soil processes with those of AMF and ECMF, which are typically studied in tree stands (e.g.Ferlian et al. 2018, Phillips et al. 2013). According to the best of our knowledge, the Mycotron experiment is the first common garden experiment that includes explicit experimentation with ERM plants and fungi in purely ERM-dominated vegetation stands, as well as in preassembled mixtures of ERM plants with AM and with ECM plants.

### Quantification of mycorrhizal fungal impacts

Controlling the level of dominance of mycorrhizal types in an ecosystem and assessing the relationship between the abundance of plants of a given mycorrhizal type and the impacts of their fungal partners on soil processes is a crucial next step in linking data on vegetation dynamics to mycorrhizal impacts on soil nutrient dynamics. Mycotron is the first experimental setup that enables such assessments. Furthermore, it allows for the investigation of interactive effects of varying proportions of AM, ECM, and ERM plants on the associated impacts of mycorrhizal fungi on soil properties.

## Conclusion

The concept of quantitative experimental research on mycorrhizal impacts on ecosystem functioning presented here establishes a benchmark for ecological experiments aimed to quantitatively unravel the mechanisms of plant–microbial interactions. The new long-term mycorrhizal experimental garden, Mycotron, addresses key knowledge gaps regarding mycorrhizal impacts on ecosystem functioning, which are essential for understanding global relationships between the dynamics of vegetation and soil processes. The proposed concept of MyFE’s, along with the insights that will be obtained through the Mycotron experiment, will broaden our understanding of fundamental ecological processes involved in the functioning of mycorrhizas, and associated ecosystem services. This is especially important now in the era of global environmental change, when humanity is in search for ecosystem restoration techniques, increasing ecosystem multifunctionality through enhanced links between soil and aboveground biodiversity.

## Supplementary Material

fiaf062_Supplemental_File
