# Supplementary material for: Towards understanding the impact of mycorrhizal fungal environments on the functioning of terrestrial ecosystems
Source: FEMS Microbiol Ecol. 2025 Jun 13;101(8):fiaf062. doi: 10.1093/femsec/fiaf062 (PMC12320777; doi:10.1093/femsec/fiaf062)
Supplement: fiaf062_Supplemental_File [file fiaf062_supplemental_file.docx]

Appendix 1: An overview of experimental conditions of each plot, and plants used.

| Plot number | condition | Mycorrhizal type | Plant species |
| --- | --- | --- | --- |
| 1 | 66% ERM 33% AM | AM | *Cotoneaster dammeri* |
|  |  | ERM | *Erica cinerea* |
|  |  | ERM | *Vaccinium vitis-idaea* |
| 2 | 100%AM | AM | *Cotoneaster dammeri* |
|  |  | AM | *Hypericum calycinum* |
|  |  | AM | *Juniperus communis* |
| 3 | 100% ERM | ERM | *Calluna vulgaris* |
|  |  | ERM | *Erica cinerea* |
|  |  | ERM | *Vaccinium vitis-idaea* |
| 4 | 66%ERM 33%ECM | ECM | *Dryas octopetala* |
|  |  | ERM | *Calluna Vulgaris* |
|  |  | ERM | *Vaccinium vitis-idaea* |
| 5 | 66%ECM 33%ERM | ECM | *Dryas octopetala* |
|  |  | ECM | *Helianthemum nummularium* |
|  |  | ERM | *Calluna vulgaris* |
| 6 | 66% AM 33% ERM | AM | *Hypericum calycinum* |
|  |  | AM | *Juniperus communis* |
|  |  | ERM | *Vaccinium vitis-idaea* |
| 7 | 33% AM 33% ECM 33% ERM | AM | *Juniperus communis* |
|  |  | ECM | *Dryas octopetala* |
|  |  | ERM | *Calluna vulgaris* |
| 8 | 66% ECM 33% AM | AM | *Juniperus communis* |
|  |  | ECM | *Halimium umbellatum* |
|  |  | ECM | *Helianthemum nummularium* |
| 9 | 66% ECM 33% ERM | ECM | *Dryas octopetala* |
|  |  | ECM | *Halimium umbetlatum* |
|  |  | ERM | *Vaccinium vitis-idaea* |
| 10 | 66% AM 33% ECM | AM | *Juniperus communis* |
|  |  | AM | *Hypericum calycinum* |
|  |  | ECM | *Helianthemum nummularium* |
| 11 | 100% ECM | ECM | *Dryas octopetala* |
|  |  | ECM | *Halimium umbellatum* |
|  |  | ECM | *Helianthemum nummularium* |
| 12 | 66%ERM 33%ECM | ECM | *Dryas octopetala* |
|  |  | ERM | *Erica cinerea* |
|  |  | ERM | *Vaccinium vitis-idaea* |
| 13 | 33%AM 33% ECM 33% ERM | AM | *Cotoneaster dammeri* |
|  |  | ECM | *Halimium umbellatum* |
|  |  | ERM | *Erica cinerea* |
| 14 | 66% AM 33% ECM | AM | *Cotoneaster dammeri* |
|  |  | AM | *Juniperus communis* |
|  |  | ECM | *Halimium umbellatum* |
| 15 | 100% ECM | ECM | *Dryas octopetala* |
|  |  | ECM | *Halimium umbellatum* |
|  |  | ECM | *Helianthemum nummularium* |
| 16 | 66% ERM 33% ECM | ECM | *Helianthemum nummularium* |
|  |  | ERM | *Calluna vulgaris* |
|  |  | ERM | *Erica cinerea* |
| 17 | 100%AM | AM | *Cotoneaster dammeri* |
|  |  | AM | *Hypericum calycinum* |
|  |  | AM | *Juniperus communis* |
| 18 | 66% ERM 33% AM | AM | *Hypericum calycinum* |
|  |  | ERM | *Calluna vulgaris* |
|  |  | ERM | *Erica cinerea* |
| 19 | 100% ERM | ERM | *Calluna vulgaris* |
|  |  | ERM | *Erica cinerea* |
|  |  | ERM | *Vaccinium vitis-idaea* |
| 20 | 66% AM 33% ERM | AM | *Cotoneaster dammeri* |
|  |  | AM | *Juniperus communis* |
|  |  | ERM | *Calluna vulgaris* |
| 21 | 100% ECM | ECM | *Dryas octopetala* |
|  |  | ECM | *Halimium umbellatum* |
|  |  | ECM | *Helianthemum nummularium* |
| 22 | 100 % ERM | ERM | *Calluna vulgaris* |
|  |  | ERM | *Erica cinerea* |
|  |  | ERM | *Vaccinium vitis-idaea* |
| 23 | 66% ECM 33% AM | AM | *Hypericum calycinum* |
|  |  | ECM | *Halimium mbellatum* |
|  |  | ECM | *Helianthemum nummularium* |
| 24 | 66%AM 33%ECM | AM | *Cotoneaster dammeri* |
|  |  | AM | *Hypericum calycinum* |
|  |  | ECM | *Helianthemum nummularium* |
| 25 | 66%ERM 33% AM | AM | *Juniperus communis* |
|  |  | ERM | *Calluna vulgaris* |
|  |  | ERM | *Erica cinerea* |
| 26 | 33% AM 33% ECM 33% ERM | AM | *Hypericum calycinum* |
|  |  | ECM | *Helianthemum nummularium* |
|  |  | ERM | *Vaccinium vitis-idaea* |
| 27 | 66% ECM 33% ERM | ECM | *Halimium umbellatum* |
|  |  | ECM | *Helianthemum nummularium* |
|  |  | ERM | *Erica cinerea* |
| 28 | 66% ECM 33% AM | AM | *Cotoneaster dammeri* |
|  |  | ECM | *Dryas octopetala* |
|  |  | ECM | *Helianthemm* |
| 29 | 66% AM 33% ECM | AM | *Cotoneaster dammeri* |
|  |  | AM | *Hypericum calycinum* |
|  |  | ECM | *Dryas octopetala* |
| 30 | 100% ERM | ERM | *Calluna vulgaris* |
|  |  | ERM | *Erica cinerea* |
|  |  | ERM | *Vaccinium vitis-idaea* |
| 31 | 100% AM | AM | *Cotoneaster dammeri* |
|  |  | AM | *Hypericum calycinum* |
|  |  | AM | *Juniperus communis* |
| 32 | 100% ECM | ECM | *Dryas octopetala* |
|  |  | ECM | *Halimium umbellatum* |
|  |  | ECM | *Helianthemum nummularium* |
| 33 | 66% ECM 33% ERM | ECM | *Dryas octopetala* |
|  |  | ECM | *Halimium umbellatum* |
|  |  | ERM | *Erica cinerea* |
| 34 | 66% ECM 33% AM | AM | *Juniperus communis* |
|  |  | ECM | *Dryas octopetala* |
|  |  | ECM | *Halimium umbellatum* |
| 35 | 100% AM | AM | *Cotoneaster dammeri* |
|  |  | AM | *Hypericum calycinum* |
|  |  | AM | *Juniperus communis* |
| 36 | 66% ERM 33% ECM | ECM | *Halimium umbellatum* |
|  |  | ERM | *Erica cinereal* |
|  |  | ERM | *Vaccinium vitis-idaea* |
| 37 | 66% AM 33% ERM | AM | *Cotoneaster dammeri* |
|  |  | AM | *Hypericum calycinum* |
|  |  | ERM | *Calluna vulgaris* |
| 38 | 33% AM 33% ECM 33% ERM | AM | *Cotoneaster dammeri* |
|  |  | ECM | *Dryas octopetala* |
|  |  | ERM | *Vaccinium vitis-idaea* |
| 39 | 66% AM 33% ERM | AM | *Hypericum calycinum* |
|  |  | AM | *Juniperus communis* |
|  |  | ERM | *Erica cinerea* |
| 40 | 66% ERM 33% AM | AM | *Cotoneaster dammeri* |
|  |  | ERM | *Calluna vulgaris* |
|  |  | ERM | *Vaccinium vitis-idaea* |
| 41 | 66%ECM 33% AM | AM | *Cotoneaster dammeri* |
|  |  | ECM | *Dryas octopetala* |
|  |  | ECM | *Halimium umbetllatum* |
| 42 | 100% AM | AM | *Cotoneaster dammeri* |
|  |  | AM | *Hypericum calycinum* |
|  |  | AM | *Juniperus communis* |
| 43 | 33% AM 33% ECM 33% ERM | AM | *Juniperus communis* |
|  |  | ECM | *Helianthemum nummularium* |
|  |  | ERM | *Erica cinerea* |
| 44 | 66% AM 33% ERM | AM | *Cotoneaster dammeri* |
|  |  | AM | *Hypericum calycinum* |
|  |  | ERM | *Erica cinerea* |
| 45 | 66% ECM 33% ERM | ECM | *Dryas octopetala* |
|  |  | ECM | *Helianthemum nummularium* |
|  |  | ERM | *Vaccinium vitis-idaea* |
| 46 | 66% ERM 33% ECM | ECM | *Helianthemum nummularium* |
|  |  | ERM | *Calluna vulgaris* |
|  |  | ERM | *Vaccinium vitis-idaea* |
| 47 | 100% ECM | ECM | *Dryas octopetala* |
|  |  | ECM | *Halimium umbellatum* |
|  |  | ECM | *Helianthemum nummularium* |
| 48 | 100% ERM | ERM | *Calluna vulgaris* |
|  |  | ERM | *Erica cinereal* |
|  |  | ERM | *Vaccinium vitis-idaea* |
| 49 | 66% AM 33% ECM | AM | *Cotoneaster dammeri* |
|  |  | AM | *Juniperus communis* |
|  |  | ECM | *Dryas octopetala* |
| 50 | 33% AM 33% ECM 33% ERM | AM | *Hypericum calycinum* |
|  |  | ECM | *Halimium umbellatum* |
|  |  | ERM | *Calluna vulgaris* |
| 51 | 66% ERM 33% AM | AM | *Juniperus communis* |
|  |  | ERM | *Calluna vulgaris* |
|  |  | ERM | *Vaccinium vitis-idaea* |
| 52 | 100% ECM | ECM | *Dryas octopetala* |
|  |  | ECM | *Halimium umbellatum* |
|  |  | ECM | *Helianthemum nummularium* |
| 53 | 66% ERM 33% AM | AM | *Hypericum calycinum* |
|  |  | ERM | *Erica cinerea* |
|  |  | ERM | *Vaccinium vitis-idaea* |
| 54 | 66% AM 33% ECM | AM | *Hypericum calycinum* |
|  |  | AM | *Juniperus communis* |
|  |  | ECM | *Halimium umbellatum* |
| 55 | 66% ECM 33% AM | AM | *Hypericum calycinum* |
|  |  | ECM | *Dryas octopetala* |
|  |  | ECM | *Helianthemum nummularium* |
| 56 | 100% AM | AM | *Cotoneaster dammeri* |
|  |  | AM | *Hypericum calycinum* |
|  |  | AM | *Juniperus communis* |
| 57 | 66% ERM 33% ECM | ECM | *Halimium umbellatum* |
|  |  | ERM | *Calluna vulgaris* |
|  |  | ERM | *Erica cinerea* |
| 58 | 66% ECM 33% ERM | ECM | *Halimium umbellatum* |
|  |  | ECM | *Helianthemum nummularium* |
|  |  | ERM | *Calluna vulgaris* |
| 59 | 100% ERM | ERM | *Calluna vulgaris* |
|  |  | ERM | *Erica cinerea* |
|  |  | ERM | *Vaccinium vitis-idaea* |
| 60 | 66% AM 33% ERM | AM | *Cotoneaster dammeri* |
|  |  | AM | *Juniperus communis* |
|  |  | ERM | *Vaccinium vitis-idaea* |
